# In Bonobos Yawn Contagion Is Higher among Kin and Friends

**DOI:** 10.1371/journal.pone.0049613

**Published:** 2012-11-14

**Authors:** Elisa Demuru, Elisabetta Palagi

**Affiliations:** 1 Dipartimento di Biologia Evolutiva e Funzionale, Università di Parma, Parma, Italy; 2 Museo di Storia Naturale e del Territorio, Università di Pisa, Calci, Pisa, Italy; 3 Istituto di Scienze e Tecnologie della Cognizione, Consiglio Nazionale delle Ricerche, Roma, Italy; Max Planck Institute for Evolutionary Anthropology, Germany, Germany

## Abstract

In humans, the distribution of yawn contagion is shaped by social closeness with strongly bonded pairs showing higher levels of contagion than weakly bonded pairs. This ethological finding led the authors to hypothesize that the phenomenon of yawn contagion may be the result of certain empathic abilities, although in their most basal form. Here, for the first time, we show the capacity of bonobos (*Pan paniscus*) to respond to yawns of conspecifics. Bonobos spontaneously yawned more frequently during resting/relaxing compared to social tension periods. The results show that yawn contagion was context independent suggesting that the probability of yawning after observing others' yawns is not affected by the propensity to engage in spontaneous yawns. As it occurs in humans, in bonobos the yawing response mostly occurred within the first minute after the perception of the stimulus. Finally, via a Linear Mixed Model we tested the effect of different variables (e.g., sex, rank, relationship quality) on yawn contagion, which increased when subjects were strongly bonded and when the triggering subject was a female. The importance of social bonding in shaping yawn contagion in bonobos, as it occurs in humans, is consistent with the hypothesis that empathy may play a role in the modulation of this phenomenon in both species. The higher frequency of yawn contagion in presence of a female as a triggering subject supports the hypothesis that adult females not only represent the relational and decisional nucleus of the bonobo society, but also that they play a key role in affecting the emotional states of others.

## Introduction

In humans (*Homo sapiens*), seeing, hearing, reading, or simply thinking about another individual yawning stimulates a similar response in the observer [1]. About 50% of human subjects yawn within a few minutes after watching a video of a yawning person [2]. Yawning can be induced in chimpanzees (*Pan troglodytes*) by observing a video of a conspecific yawning [3], [4], even when the “conspecific” is a 3D-animated chimpanzee [5]. As for monkeys, yawn contagion has been demonstrated via an observational, highly standardized approach in gelada baboons (*Theropithecus gelada*) living under natural conditions [6]. Outside the Primate Order, there have been some attempts to investigate this phenomenon also in dogs (*Canis familiaris*). The different authors, who approached the topic in this species, gained contrasting findings even on its mere presence [7]–[10]. Hence, if yawn contagion is present in dogs is still an open argument.

Since most yawn events occur in social contexts, it has been hypothesized that the infectiousness of yawning may be linked to emotional arousal [11] and may have a communicative function (the hypothesis states that yawn contagion is a non-verbal form of communication that synchronizes the behavior of a group, for an extensive review see [12]).

The ability to share emotional states, a phenomenon known as empathy, relies on a perception-action mechanism and is essential for successful social interactions [13]. During the observation of a facial expression, the observer involuntary re-enacts the same motor pattern by recruiting neural mechanisms that concurrently activate the same affective state associated with that specific facial expression [13]–[15]. Some recent studies suggest that yawn contagion is based on a similar mechanism and could reflect a basic form of empathy, which can be tentatively defined as the capacity to catch and feel in an unconscious and automatic way an emotional state expressed by another individual [6], [16], [17]. The linkage between yawn contagion and empathy in humans is supported by clinical, psychological, neurobiological, and ethological clues. Subjects suffering from empathy-related disorders, such as autism or schizophrenia, show lower levels of yawn contagion [18]–[21]; whereas, subjects obtaining higher scores in questionnaires evaluating empathy and mental state attribution show higher rates of yawn contagion [22]. From a neurobiological perspective, several neuroimaging studies support the empathic basis of yawn contagion [23]–[25]. Viewing someone yawning activates the posterior cingulate and precuneus, areas known to be part of empathy networks [23]. The relationship between yawn contagion and emotional involvement is also underlined by the activation of the ventromedial prefrontal cortex, a region involved in the empathic processes [26]–[28] and also associated with the propensity to respond to a yawn stimulus [25]. Therefore, although evidence is still under debate [24], mirror neurons [28], [29] might be recruited for yawn contagion. Mirror neurons fire when an animal performs an action, as well as when it perceives another animal performing the same action [30], [31]. Accordingly, the mirror neuron system is important for action understanding, a prerequisite for empathy [30]–[32], and may be part of the neural network underlying imitative actions [33]–[35].

From a behavioral point of view, the positive correlation between yawn contagion and social bonding, already demonstrated in geladas [6] and humans [16], fits the hypothesis that a link between yawn contagion and empathy may exist. The perception-action model predicts that in social species, empathy is biased toward individuals who are more similar, familiar, or socially closer [13]. In our species, yawn contagion and the degree of emotional closeness are positively correlated such that occurrence, frequency, and latency of the response are distributed according to an empathic gradient [13], which follows the scheme: kin>close friends>acquaintances>strangers [16].

The only study on the frequency of yawn contagion in non-human apes, although based on an A then B design, indicates that it differs between familiar and unfamiliar subjects [36]. Even though they attended more to the videos of unfamiliar subjects, chimpanzees yawned more when watching yawns performed by familiar than unfamiliar individuals, suggesting an ingroup-outgroup bias in contagious yawning. The authors discussed the finding as further empirical evidence about contagious yawning as a measure of empathy. However, to our knowledge, no behavioral systematic study investigated the linkage of yawn contagion and social closeness among apes living in the same group and tested under natural conditions.

Via a standardized observational approach we investigated yawn contagion and its distribution in a captive group of bonobos (*Pan paniscus*). Bonobos are defined by the majority of the authors [37]–[42] as a highly prosocial and tolerant species, characterized by strong affinitive relationships even among unrelated subjects [37]–[39]. They show a vast repertoire of social behaviors such as play [40], socio-sexual interactions [38], and consolation [37], aimed at increasing the cohesiveness among group members, especially among females (female bonded society) [41], [42]. Moreover, in a recent study comparing the neural circuitry implicated in social cognition in the two *Pan* species, Rilling et al. [43] found that bonobos, compared to chimpanzees, have more developed cortical brain areas involved in perceiving distress in both oneself and others, an emotional state underpinning empathic abilities. Compared to chimpanzees, bonobos also have a larger pathway linking the amygdala with the ventral anterior cingulate cortex, a pathway implicated in both top–down control of aggressive impulses, as well as bottom–up biases against harming others [43]. As a whole, such neurobiological findings strongly support bonobos' empathic sensitivity and propensity to prosociality. For all these reasons, the bonobo is a good model species to test some hypotheses about the possible linkage between yawn contagion and certain empathic abilities, although in their most basal form.

Here, we report that yawning is contagious in *Pan paniscus* and that yawn contagion is independent from the social context and from the amount of spontaneous yawns performed. Moreover, we found that contagion was higher when the triggering subject was a female, as predicted for a female bonded society. Finally, our data show that in bonobos yawn contagion distribution reflects what has been found in humans [16], with kin and friend dyads showing the highest level of yawn contagion.

## Results

We collected behavioral data during 3 months of observation (August–October 2009) on all the subjects of the Apenheul colony, which was composed by 12 bonobos (2 adult males, 6 adult females, 4 infants). In the observational period we recorded 1,260 yawns by using the all-occurrences sampling (502 h). Since it has been demonstrated that both human [44]–[45] and chimpanzee infants [3] are not infected by others' yawn, we limited the analysis to adults obtaining a final dataset of 1,125 yawns.

The concurrent presence of two observers over the whole period of data collection permitted the recording of the identity of each group member that was in visual and/or auditory contact with the yawner. Consequently, we could assess which individuals did not perceive the yawn.

**Figure 1:**
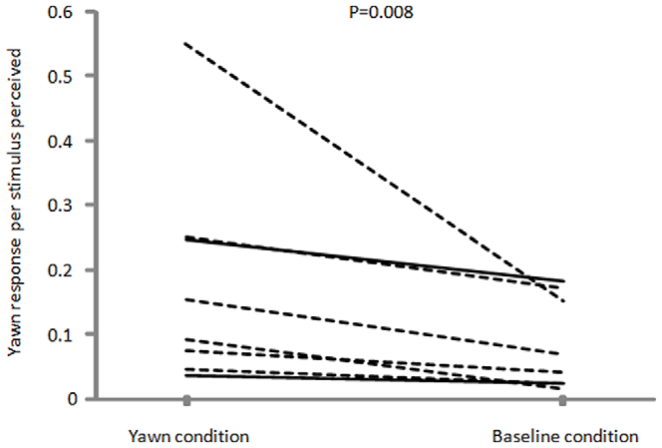
Yawn contagion in bonobos: individual frequency of yawns in presence (yawn condition) and in absence (baseline condition) of the stimulus (triggering yawn). Dotted lines represent females, full lines represent males.

**Figure 2:**
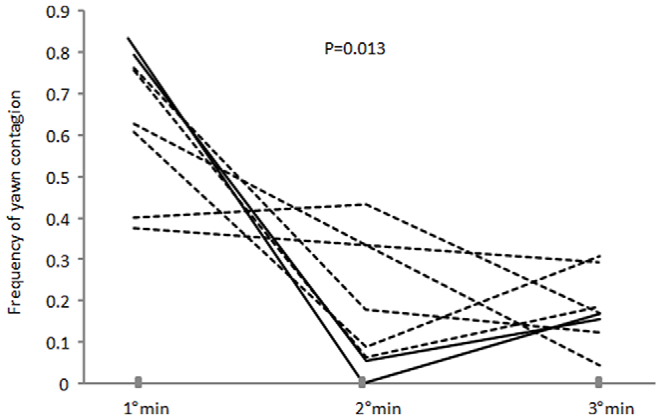
Individual frequency of yawn contagion as a function of the minute of observation. Dotted lines represent females, full lines represent males.

After the first yawn event (stimulus) emitted by an individual (hereafter, the triggering subject), we observed all subjects for 3 minutes and recorded if they yawned or not. The subjects were divided into two groups: those who perceived the yawn stimulus (yawn condition) and those who did not (baseline condition). In the yawn condition, when it was not possible to attribute the response univocally to a given individual (more than one subject yawned within the 3 min preceding the response), that response was excluded from the analysis. So we obtained a total of 295 yawns deriving from contagion. The frequency of yawns was significantly higher in the yawn condition compared to the baseline condition (Exact Wilcoxon's T = 0, ties = 0, n = 8, P = 0.008) (Figure 1). We then calculated the individual frequency of yawn contagion during each minute of observation and we found that yawn contagion mostly occurred within the first minute (Exact Friedman test χ^2^, = 8.07, df = 2, N = 8, p = 0.013) (Figure 2).

**Figure 3:**
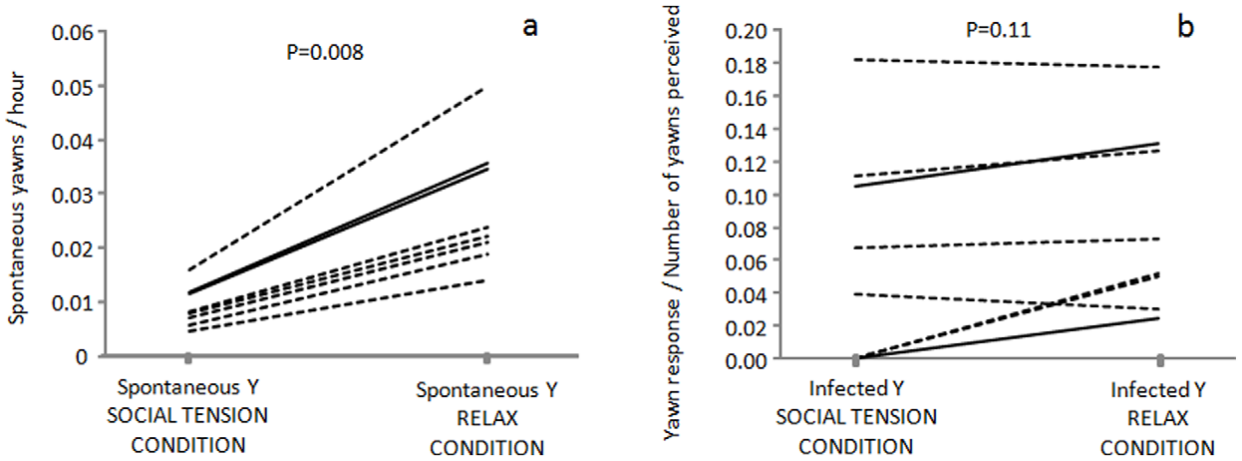
Individual hourly frequency of spontaneous yawns (a) and individual yawn response per number of yawns perceived (b) occurring under the two different social conditions: social tension (post-conflict, captive management, pre-feeding, and feeding) and relax (all the remaining periods of time) (see **Methods** **for definitions).** Dotted lines represent females, full lines represent males.

All the yawn events not preceded by a yawn stimulus in the previous 3 minutes were labeled as spontaneous. We recorded all the spontaneous yawns occurring under two different social conditions: social tension (post-conflict, captive management, pre-feeding, and feeding) and relax (all the remaining periods of time) (see Methods for definitions). We found that spontaneous yawning occurred more frequently in the relax condition (Exact Wilcoxon's T = 0, ties = 0, n = 8, P = 0.008) (Figure 3a). The same result was not found when considering the infected yawns, whose distribution did not differ in the two conditions (Exact Wilcoxon's T = 2, ties = 0, n = 7, P = 0.11) (Figure 3b). One female was excluded from this last analysis because she perceived less than 6 yawns as stimulus during the social tension condition.

**Figure 4:**
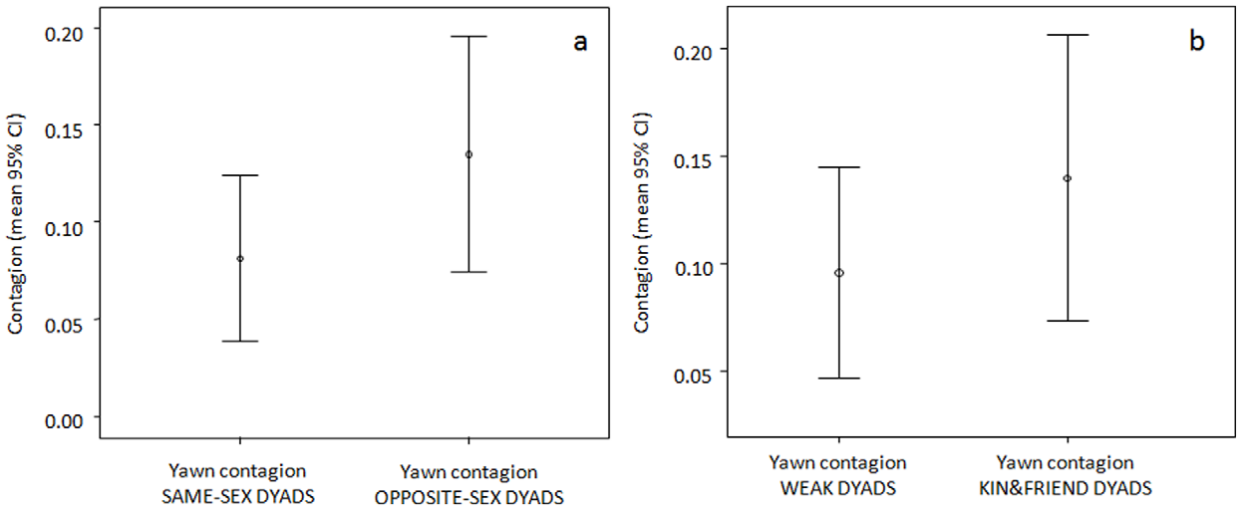
Frequency of yawn contagion as a function of the sex of the subjects involved (a) and of the relationship quality of the dyads involved (b).

Via a Linear Mixed Model (LMM) we assessed which variables might explain the differences in the frequency of yawn contagion during the first minute (dependent variable). Triggering subject's and responder's gender and rank, sex combination, and social bonding were entered as fixed factors (Table 1 and Methods for definitions). This analysis involved only those dyads (n = 48) where yawn contagion had occurred and in which each of the two subjects had at least 6 opportunities to see the other's yawn. Sex combination, social bonding, and triggering subject's gender remained in the best model (AICc = −55.96). They positively affected the frequency of yawn contagion, which increased when the triggering subject and the responder belonged to different genders (Figure 4a, same-sex dyads: mean 0.057±0.04 SE; opposite-sex dyads: mean 0.145±0.03 SE) and alongside the tightness of the social bonding (Figure 4b, kin&friends: mean 0.134±0.03 SE; weakly bonded: mean 0.07±0.03 SE). Moreover, the frequency of yawn contagion tended to be higher when the triggering subject was a female (female triggering subject: mean 0.144±0.03 SE; male triggering subject: 0.057±0.04 SE) (see Table 2 for statistics).

**Table 1:** Description of the variables used in LMM analysis.

NAME OF VARIABLES	TYPE OF VARIABLES
**DEPENDENT VARIABLE**	
Frequency of yawn contagion	Scale (positive integer values)
**FIXED EXPLANATORY VARIABLES**	
**Individual characteristics**	
Rank	Ordinal (1 = high; 2 = low)
Gender	Ordinal, dichotomous (1 = female; 0 = male)
Sex combination	Ordinal, dichotomous (0 = same sex; 1 = different sex)
**Relationship characteristics**	
Kinship & Affiliation	Ordinal, dichotomous (1 = kin&friends; 0 = weakly bonded)
**RANDOM VARIABLES**	
Trigger's identity	Nominal
Responder's identity	Nominal

**Table 2:** Best LMM explaining the occurrence of yawn contagion within the first minute (AICc = −55.96).

	Numerator df	Denominator df	F	Significance level
Intercept	1	5.00	12.31	0.017
**FIXED FACTORS**				
Trigger's gender	1	6.35	4.02	0.079
Sex combination	1	34.10	5.57	0.024
Social bonding	1	39.20	4.87	0.033
**RANDOM FACTORS**	**Variance**	**SE**		
Trigger's identity	0.0004	0.0017		
Responder's identity	0.0039	0.0031		

df: degrees of freedom; SE: standard error.

## Discussion

Our data show, for the first time, that contagious yawning is also present in another great ape species, *Pan paniscus* (Figure 1). The study, conducted within a naturalistic framework, permitted us to shed light on some interesting aspects of the yawn contagion modality in this species.

In bonobos the yawing response mostly occurred within the first minute after the perception of the yawn stimulus (Figure 2). This response latency is similar to that observed in humans [16] but differs from that of gelada baboons, in which the yawn contagion typically peaked in the second minute after the triggering stimulus [6]. As a result of phylogenetic inertia, the brain of non-human apes shows more elements of similarity with that of humans than with that of cercopithecoids [46]. Even though the interpretation of this finding has to be taken with caution, the similarity of bonobo and human yawn response latency might reflect the similarity of the neural pathways underpinning yawn contagion in the two species.

Spontaneous yawns were more frequent when bonobos were free from environmental and social stressors (relax context) (Figure 3a), but yawn contagion was context independent (Figure 3b), thus suggesting that the probability of yawning after observing others' yawns is not affected by the propensity to engage in spontaneous yawns. Both in humans and other animals, spontaneous and contagious yawning may be driven by different mechanisms [47]. Spontaneous yawning may be more strictly linked to physiological factors such as respiratory activity [48], [49], thermoregulation [50], changes in vigilance/arousal levels [51]–[53], and sleep/wake transitions [54]–[56]. When a triggering stimulus is present, the yawn response seems to be disentangled from physiological/contextual conditions (social tension vs relax). This finding supports the communicative hypothesis of yawn contagion [6], [12], [16], [36].

In bonobos, yawn contagion increased with social closeness (Figure 4b), thus mirroring what found in *Homo sapiens*, in which emotional bonding and kinship modulate yawn contagion as well [16]. From an adaptive point of view, yawn contagion (as other forms of unconscious mimicry, see [17] for an extensive review) can aid social groups to synchronize their activities (communicative hypothesis of yawn contagion) [2]. Yet, yawn contagion, compared to other forms of unconscious mimicry, seems to be enriched by an emotional component [17], as it is suggested by its higher frequency between emotionally bonded subjects.

Although the argument is still under debate [57], bonobos are generally recognized by a wide array of authors as one of the most prosocial and tolerant non-human primates [39], [42], [58]–[62]. Roberts and Strayer [63] found that emotional expressiveness and anger are important predictors of empathy for school-age children, and that empathy strongly predicted prosocial behaviors aggregated across methods and sources. As it has been done for humans, a further hint supporting a possible link between yawn contagion and empathy in apes could arise from studies (through both naturalistic and experimental approaches) that correlate yawn contagion to prosocial behaviours, which are hypothesized to be empathy-related (e.g. consolation [37], [64], [65] and targeted helping [66], [67]). Moreover, some authors [68], [69] demonstrated that in humans the same mechanisms that cause empathy to enhance prosocial behaviors should also cause it to inhibit aggression and the expression of anger. In this perspective, it would be interesting to verify if, in the great apes, the subjects more inclined to be infected by others' yawns are also more inhibited to engage in aggressive behavior.

Some authors suggested that an attention bias (with observers paying closer attention to familiar subjects rather than to unfamiliar ones) could affect the yawning response distribution [70]. Since it is extremely difficult to quantify the attention level of a subject under both experimental and naturalistic conditions, we cannot exclude that an attention bias might affect the studies on yawn contagion. The only variable that can be controlled is the unambiguous possibility to perceive the stimulus, for that reason in this kind of research the analysis has to be strictly limited only to those events that are surely perceived. Yet, some clues indicate that heightened arousal (degree of physiological responsivity relative to a baseline) is normally detected in response to novelty, whereas diminished arousal is observed in response to perceived familiarity (habituation process), an evolutionary adaptation, which has been interpreted by some authors as a mechanism to avoid the overloading of the attentional system [71]. Moreover, it has been recently demonstrated that in patients with unilateral destruction of the visual cortex (cortical blindness), “*a passive exposure to unseen expressions evoked faster facial reactions and higher arousal compared with seen stimuli, therefore indicating that emotional contagion occurs also when the triggering stimulus cannot be consciously perceived*” [72, p. 17661].

The evidence that yawn contagion is shaped by social closeness is consistent with the hypothesis that this phenomenon is a form of emotional contagion relying on a basic form of empathy. This association, already hypothesized for geladas [6] and humans [16], two phylogenetically distant species within the Primate Order, suggests that it could be either deeply rooted in the evolutionary history of the *taxon* or the outcome of convergence. Our finding on a non-human ape, the bonobo, supports the idea that the link between yawn contagion and a basic from of empathy is not due to evolutionary convergence but it is, instead, a common ancestral trait shared by monkeys and apes, including humans.

The higher frequency of yawn contagion between individuals belonging to different genders (Figure 4a) and in presence of a female as a triggering subject suggests that bonobo males are more affectively reactive towards females, who constitute the core of social groups [73]. Massen and co-workers [4] recently demonstrated that, in chimpanzees, male yawns were far more contagious than those of females. In addition, individuals of the dominant and bonded sex (i.e. males in *Pan troglodytes*, [74]) infected each other at the highest levels. Even though our findings have to be taken with caution due to the small sample size of adult males, in bonobos yawn contagion appears to support the hypothesis that adult females not only represent the relational and decisional nucleus of the society [38], but also that they play a key role in affecting the emotional states of others.

In conclusion, even though we are still far from a clear demonstration of a linkage between yawn contagion and empathy, the importance of social bonds in shaping bonobo yawn contagion seems to support the hypothesis that a basic form of empathy can play a role in the modulation of this phenomenon. As for *Homo sapiens*, yawn contagion in *Pan paniscus* is amplified when an emotional involvement is present, as it occurs among kin and friends.

## Methods

### Ethics statement

This study was approved by University of Pisa (Animal Care and Use board). Since the study was purely observational the committee waived the need for a permit. The study was conducted with no manipulation of animals.

### The study species

The bonobo (*Pan paniscus*) is one of the closest living relatives to humans [75]. This great ape shares many basic features with humans [76]. They have a high level of behavioral flexibility and individuals aggregate into cohesive multimale-multifemale societies [41]. Bonobos live in communities, whose members form temporary parties that vary in size and composition [42], [77]. The species is characterized by male philopatry and female dispersal [41]. Bonobos show a high level of female cohesion reached also by i) an intense socio-sexual activity, agonistic support, and play; ii) an absence of male dominance; and iii) a strong tendency of feeding priority for females [38].

### The study group

Behavioral data were collected during 3 months of observation (August–October 2009) on a group of *Pan paniscus* housed in the Apenheul Primate Park (Apeldoorn, The Netherlands), first established in 1998. During data collection, the colony was composed of 12 individuals (2 adult males, 6 adult females, and 4 immature subjects). The animals were housed in an enclosure with both an indoor and outdoor facility (about 230 m^2^ and 5,000 m^2^, respectively) and could move freely from the indoor to the outdoor enclosure after the first feeding session (at about 9:00 AM), and received abundant food (pellets, vegetables, fruits, rice and nuts, that were scattered on the ground) three times a day at 9:00 AM, 12:45 PM, and 5:00 PM. Water was available *ad libitum* and environmental enrichments were provided in the form of fresh branches, rice, and nuts scattered on the grass to encourage foraging activity, and renewal of the equipment in the indoor facility. Sometimes seeds and a wooden block with holes filled with honey, syrup were also furnished. No stereotypic or aberrant behaviors were observed during the entire period of data collection.

Daily observations covered a 6-hr period, encompassing both morning and afternoon. Data were collected by two observers (one of them was E. D.) by using a voice-recorder, and the records were then computer transcribed on database sheets. For the data collection a rigorous and repeatable observation protocol was developed by E. P. before commencing systematic data collection, the two observers underwent a training period (the trainer was E. P.) during which they followed the same focal animals simultaneously and then compared data. The training was considered completed when the observations of the two observers matched in 95% of cases [78]. The training period lasted approximately 50 h of focal sampling. As this was part of a long-term project, a wide array of data regarding various social behaviors and contexts was collected according to a blind coding protocol, in which observers were not aware of the hypotheses and predictions that would have been tested. The social ethogram used was based on the ethograms formulated by Kano [79], Enomoto [80] and de Waal [81] and developed by E. P. on the basis of previous observations performed on several bonobo colonies.

Under some conditions, it is possible to record all occurrences of certain classes of behaviors in all members of a group during every observation period. Such records are generally possible when observational conditions are excellent, the behaviors are sufficiently ‘attention-attracting’, and the behavioral events never occur too frequently. As in our case all these conditions were met, it was possible to use the all occurrences sampling technique (about 502 h) [82] to collect any yawning event.

By focal animal sampling (25 h of observation per subject), we were able to record all the contact sitting, grooming, and food-sharing sessions performed by each focal animal with any other group member. Each subject was followed every day (each focal lasted 30 min) and at different times to obtain data covering the entire day in balanced proportions as much as possible.

### Operational definitions and statistics

Social bonds were determined on two levels: kinship and affiliation. Kinship was based on maternal lineages, and only mother-offspring were considered to be related individuals. The affiliation levels between dyads were categorized using a combined measure of three behaviors collected during focals (i.e., contact sitting, grooming, and food sharing) and calculating the quartile points of dyadic scores for each focal individual. Only dyads with scores in the top quartile were considered to have a strong affinitive relationship (friends). Since our sample was characterized by only 3 kin dyads (mother-offspring, r = 0.5), we decided to create a category including both kin and friends (kin&friends; dyads n = 12). All the other dyads were labeled as weakly bonded.

Individuals' ranking position was assessed by entering decided conflicts into a winner/loser socio-matrix. Such socio-matrix was reordered via Matman 1.0 and two rank levels were recognized: high (if an animal's rank fell into the upper rank quartile) and low (if an animal's rank fell outside the upper rank quartile).

We categorized our observations into two different social contexts: social tension and relax. The social tension context included post-conflict periods (10 min after an agonistic interaction), captive management activities (from the beginning of the operations till 20 min after the keepers left the enclosure), pre-feeding (10 min before food distribution), and feeding (10 min after the food distribution). The relax condition included all the remaining periods of observation time.

Owing to the small sample size (n = 8), the comparisons (yawn contagion in yawn *vs* baseline conditions; frequency of spontaneous and infected yawns in the social tension *vs* relax condition) were run via the non-parametric Wilcoxon's test. The Friedman's test (k = 3) was used to assess the time latency of yawn contagion. Sample size and animals differed across tests because in each analysis we could include only individuals meeting all conditions [83].

To examine the effect of different variables on the frequency of yawn contagion, a Linear Mixed Model (LMM) was run. The dependent scale variable was the relative frequency of yawn contagion by the responder measured as the number of times such responder had yawned after a given triggering subject's yawn normalized on the number of occasions (minimum 6).

In all analyses, triggering subject and observers' identities were entered as random factors (nominal variables). We tested models for each combination involving the variables of interest (Table 1), spanning from a single-variable model to a model including all the fixed factors (full model). To select the best model, we used the Akaike's Corrected Information Criterion (AICc), a measure for comparing mixed models based on the −2 (Restricted) log likelihood. The AICc corrects the Akaike's Information Criterion (AIC) for small sample sizes. As the sample size increases, the AICc converges to AIC. The model with a lower value of AIC was considered to be the best model.
